# Functional impairment triggered by altertoxin II (ATXII) in intestinal cells in vitro: cross-talk between cytotoxicity and mechanotransduction

**DOI:** 10.1007/s00204-018-2317-6

**Published:** 2018-10-01

**Authors:** Giorgia Del Favero, Ronita Zaharescu, Doris Marko

**Affiliations:** Department of Food Chemistry and Toxicology, Faculty of Chemistry, University of Vienna, Währingerstr. 38-40, 1090 Vienna, Austria

**Keywords:** Intestinal cells, Altertoxin II (ATXII), Migration, Shear stress, Membrane fluidity

## Abstract

Intestinal cells are able to continuously integrate response to multiple stimuli/stressors; these include the concomitant activation of “chemically driven” pathways, of paramount importance in the response to toxicants, as well as physical stimulation derived from motility. Altertoxin II (ATXII, 0.1, 1 and 10 µM), a mycotoxin produced by the food contaminant fungus *Alternaria alternata* was studied in HT-29 intestinal adenocarcinoma cells and in non-transformed intestinal epithelial cells, HCEC. One-hour incubation with ATXII was sufficient to trigger irreversible cytotoxicity in both cell types, as well as to modify cellular responses to concomitant pro-oxidant challenge (H_2_O_2_, 100–500 µM, DCF-DA assay) suggesting that even relatively short-time exposure of the intestinal cells could be sufficient to alter their functionality. Combination of ATXII (1 µM) with physical stimulation typical of the intestinal compartment (shear stress) revealed differential response of tumor-derived epithelial cells HT-29 in comparison to HCEC, in particular in the localization of the transcription factor Nrf2 (NF-E2-related factor 2). Moreover, ATXII reduced the migratory potential of HCEC as well as their membrane fluidity, but had no respective impact on HT-29 cells. Taken together, ATXII appeared to alter predominantly membrane functionality in HCEC thus hampering crucial functions for cellular motility/turnover, as well as barrier function of healthy intestinal cells and had very limited activity on the tumor counterparts.

## Introduction

In the gastrointestinal compartment, cells are continuously challenged by the presence of food constituents and contaminants that might interact in a very complex environment. In fact, in addition to food-borne substances, luminal cells experience strong mechanical stimulation due to the peristaltic movement of the intestinal segments (Basson 2003). Moreover, continuous cellular turnover requires cell movement and migration that ultimately results in a highly dynamic milieu. From the toxicological perspective, this can be translated into the need for single cells to face at the same time nutrients, toxicants, and integrate the physiological responses as well as toxicity pathways together with mechanotransduction (Gayer and Basson 2009). Similarly, cytotoxicity might result in a severe impairment of correct cellular function, but also in the inability to respond promptly and dynamically to the surrounding environment. In this respect, the difference in sensitivity between cancer cells and healthy epithelium is still largely an open question. It is well known that the majority of the cell models can undergo morphological differentiation in the presence of physical stimulation that mimics more physiological conditions (Goodwin et al. 1992) and the development of in vitro models that integrate the biomechanical stimulation into cell culture is an approach that is gaining more and more relevance (Kim et al. 2012). However, the reactivity and the biological responses of different cell types to physical–chemical challenges are far from being completely understood.

Among food contaminants, mycotoxins represent an always renewing challenge; in fact, especially for the so-called “emerging mycotoxins”, thorough hazard characterization is a prerequisite for the correct application of the regulatory actions necessary to ensure food safety and appropriate protection of the consumers (Gruber-Dorninger et al. 2016). As a result, many compounds can enter the food chain simply because they are not regulated or because they contaminate the food commodities in domestic context. Among the non-regulated mycotoxins, there are a large number of the secondary metabolites from *Alternaria alternata* molds. *Alternaria alternata* proliferates on food items and can contaminate commodities which may also reach the market (Walravens et al. 2016). *Alternaria* mycotoxins can differ greatly in structure and their biological targets span from the regulation of DNA topology to the estrogenic cascade (Aichinger et al. 2017; Jarolim et al. 2016; Lehmann et al. 2006; Vejdovszky et al. 2017a). Among these, the perylene quinone type mycotoxin altertoxin II (ATXII) is one of the more potent ones with respect to genotoxicity (Fleck et al. 2016; Pahlke et al. 2016; Schwarz et al. 2012). ATXII is well known to be formed by *Alternaria alternata* strains in vitro, i.e., in rice-based culture (Schwarz et al. 2012; Zwickel et al. 2016a). However, data concerning its occurrence in food and feed are limited and great effort is continuously devoted to the development/optimization of analytical methods for its detection (Puntscher et al. 2018; Zwickel et al. 2016b). To add complexity to this scenario, actual data indicate that ATXII may promptly react with food constituents (Aichinger et al. 2018). The fate of ATXII during food production is still unknown and currently subject to intensive research, thus still hampering any reliable exposure estimation. Applied in mammalian cell culture, ATXII triggers genotoxic and mutagenic damage (Fleck et al. 2012; Pahlke et al. 2016; Schwarz et al. 2012), enhances intracellular ROS (reactive oxygen species) levels and activates the redox-sensitive Nrf2/ARE pathway (Jarolim et al. 2017; Pahlke et al. 2016). In spite of that, it was recently described that the toxin may be rapidly and efficiently biotransformed in differentiated Caco2 cells (Fleck et al. 2014a). This suggests, on the one hand, a very limited systemic absorption/bioavailability of the parent molecule and on the other hand, that the epithelium of the gastrointestinal tract can be considered a very relevant target for this compound. At the cellular level, cell membrane represents the interface of the cells with the external environment, and as such, it is a crucial player regulating cellular contact with xenobiotics, as well as being the first “mechanosensor”. Accordingly, there is increasing proof of evidence that in addition of providing a “cellular-envelope” and structural support, the membrane plays an essential role in the definition of the cellular interaction with the external environment and actively sustains the cytoskeleton in crucial cell functions such as migration and adhesion (Ayee et al. 2017; Blanchard and Busik 2017). Accordingly, the reactivity of HT-29 intestinal adenocarcinoma cells was compared to non-transformed intestinal epithelial cells, HCEC (Roig et al. 2010) to explore the differential sensitivity of tumorigenic and non-tumorigenic cell types. ATXII was used to study in tumorigenic and non-transformed cells the delicate interplay between food-borne toxicants, physical forces and mechanotransduction at intestinal level. The impact of ATXII on intestinal cells was evaluated in terms of motility, with a particular focus on the capability of intestinal cells to migrate and to respond to shear stress, which are essential prerequisites for the correct intestinal function (Bianco et al. 2012).

## Materials and methods

### Cell culture

The human colon adenocarcinoma cell line HT-29 was originally acquired from ATCC. According to the instruction of the supplier, cells were cultivated in DMEM supplemented with 10% fetal bovine serum (FBS) and 1% penicillin/streptomycin (P/S, 50 U/ml). HCEC (HCEC-1CT) were kindly provided by Prof. Jerry W. Shay (UT Southwestern Medical Center, Dallas, TX, USA) and cultivated as previously described (Khare et al. 2015; Warth et al. 2016). Briefly, cells were kept in high-glucose DMEM combined with 10X medium 199 (2%) and supplemented with cosmic calf serum (2%), HEPES 20 mM, gentamycin (50 µg/ml), insulin–transferrin–selenium-G supplement (10 µl/ml), recombinant human EGF (20 ng/ml), and hydrocortisone (1 µg/ml). Cell culture media and supplements were purchased from GIBCO Invitrogen (Karlsruhe, Germany), Lonza Group Ltd (Basel, Switzerland), Sigma-Aldrich Chemie GmbH (Munich, Germany) and Sarstedt AG&Co (Nuembrecht, Germany). Both cell lines were cultivated in humidified incubators at 37 °C and 5% CO_2_ and regularly tested for the absence of mycoplasma contamination.

### Cytotoxicity assay

Sulforhodamine B (SRB) assay was chosen to verify the cytotoxic potential of ATXII [isolated and purified as previously described (Jarolim et al. 2017)]. SRB experiments were performed according to the protocol of Skehan and collaborators (Skehan et al. 1990). Accordingly, at the end of the incubation (ATXII 0.1–10 µM; solvent controls 0.1% or 0.2% DMSO), HT-29 and HCEC were rinsed with PBS, fixed with 50% trichloroacetic acid (TCA; 30 min, 4 °C), dried and stained with SRB solution (0.4% w/w diluted in 1% acetic acid, 1 h, RT). Cells were then rinsed with diluted acetic acid (1%) and bi-distilled water. Protein-bound SRB reagent was diluted with Tris (10 mM) and absorbance was measured at 570 nm with a Victor^3^V 1420 Multilabel Counter Plate Reader (PerkinElmer, Waltham, USA). Data are presented as the means ± standard error of the mean (SE) of at least three independent cell preparations measured in technical triplicates. Statistical analysis was performed with Origin Pro 9.1G (OriginLab, Northampton, USA) applying one-way ANOVA with Fisher test for pairwise comparison (threshold value *p* < 0.05).

### Measurement of intracellular reactive oxygen species (ROS)

For the measurement of the intracellular ROS after exposure to ATXII and in relation to the integrity of the barrier function of the epithelium, cells were pre-incubated with ATXII (0.1 and 1 µM) for 1 h and subsequently with 50 µM of DCF-DA (2′,7′-dichlorofluorescein diacetate, 100 µl, 30 min, 37 °C). Afterwards, the DCF-DA containing solution was removed, cells were washed with PBS and challenged with H_2_O_2_ 100 µM and 500 µM. Measurements were carried out in Live Cell Imaging Solution measuring ex./em. 480 nm/520 nm, respectively. Fluorescence was measured with a multi-mode microplate reader Cytation3 Imaging Multi-Mode Reader (BioTek, Winooski, VT, USA). At least three independent biological replicates were performed in technical quadruplicates. Data are presented as the means ± SE and the statistical analysis was performed with Origin Pro 9.1G (OriginLab, Northampton, USA) applying one-way ANOVA with Fisher test for pairwise comparison (threshold value *p* < 0.05).

### Shear stress experiments

Shear stress experiments were performed with the Ibidi Pump System Quad (Ibidi GmbH, Martinsried, Germany). Cells were seeded in µ-Slides (poly-l-lysine coated, Ibidi GmbH, Martinsried, Germany) and allowed to settle for 48 h. For the performance of the experiments, cells were stimulated for 1 h with a shear stimulation of 1 dyn/cm^2^ (0.1 Pa). Incubation of HT-29 cells and HCEC was performed in parallel, with or without toxin. Moreover, incubation of the cells in static environment was also performed. At the end of the stimulation protocol, cells were either imaged or immediately fixed with pre-warmed formaldehyde (3.7% in PBS) for the performance of immunofluorescence experiments.

### Confocal microscopy

Imaging experiments were performed directly after biomechanical stimulation with shear stress or at the end of the incubation with the toxins. For classical immunofluorescence analysis, cells were seeded in eight-well microscopy slides (Falcon, Corning, New York, USA) or in Ibidi µ-Slides (Ibidi GmbH, Martinsried, Germany). After 48 h, cells were incubated with ATXII or solvent control (0.1% DMSO) according to the experimental protocol. At the end of the experiments, cells were either rinsed with PBS, stained for plasma membrane (CellMask™ Deep Red Plasma membrane Staining, dil. 1:1000 with or without Hoechst 33258, dil. 1:1000) and directly imaged, or fixed and further processed (formaldehyde 3.7% in PBS, 15 min, 37 °C). Permeabilization was obtained with Triton-X (0.2% in PBS, 10 min, RT), and unspecific reactive sites were blocked with donkey serum (2% in PBS, 1 h, RT). For the detection of Nrf2, an antiNrf2 rabbit polyclonal antibody (sc-722) from Santa Cruz Biotechnology (Heidelberg, Germany) was used. Immunodetection was performed with fluorescent label Alexa Fluor 568 Donkey Anti-Rabbit (A10042; dilution 1:1000). Actin cytoskeleton was visualized with Alexa Fluor™ 488 Phalloidin (Molecular Probes, Life Technologies, Thermo Fisher Scientific, Waltham, USA). After removal of secondary antibodies, slides were post-fixed with 3.7% formaldehyde (10 min, RT) and 100 mM glycine was used to mask reactive sites. Images were acquired with a confocal LSM Zeiss 710 equipped with ELYRA PS. 1 system using a Plan Apochromat 63X/1.4 oil objective (zoom 1.5) and an Andor iXon 897 (EMCCD) camera. Images were analyzed with the software Zen Zeiss 2012 and quantification was performed with *n* > 30 cells from a minimum of six optical fields obtained from at least 2/3 independent cell preparations. Statistical analysis was performed with Origin Pro 9.1G (OriginLab, Northampton, USA) applying Student’s *t* test (threshold values *p* < 0.05).

### Migration assay

For the performance of the migration assay cells were seeded in Culture-Insert 4 Well in µ-Dish 35 mm, high (Ibidi GmbH, Martinsried, Germany). Cells were allowed to grow to confluency in the wells and incubated for 1 h with 1 µM ATXII or 0.1% DMSO for the controls. At the end of the incubation, toxin-containing medium was removed, cells were rinsed with pre-warmed PBS and the insert was removed and images were acquired to obtain the initial images (time 0). Afterwards, cell type-specific complete medium was replaced. Images were acquired at differential time points and 8 h was chosen for the performance of the quantitative evaluation. Live cell imaging was performed in Live Cell Imaging Solution (Molecular Probes, Life Technologies, Thermo Fisher Scientific, Waltham, USA) using the Cytation3 Imaging Multi-Mode Reader (BioTek, Winooski, VT, USA). Image analysis was performed with Photoshop CC2015, overlapping directly the sequential images and calculating the percentage of area covered by the cells. Data are means ± SE obtained from minimum three independent cell preparations and analyzed with Origin Pro 9.1G (OriginLab, Northampton, USA) applying Mann–Whitney test (threshold values *p* < 0.05).

### Membrane fluidity assay

The membrane fluidity of HT-29 cells and HCEC was measured according to the protocol described from Zhang and co-workers with slight modifications (Zhang et al. 2011). Cells were pre-incubated with 1-pyrenedecanoic acid (PDA; Sigma-Aldrich, Merck KGaA, Darmstadt, Germany; 37 °C, 1 h) and afterwards stimulated with ATXII or solvent controls. Cholesterol complexing agent methyl-β-cyclodextrin (10 µM, MβCD) was added as comparison. Quantification of the dye in monomeric form was performed at 375 nm and in excimeric form at 470 nm (excitation 344 nm) using the Cytation3 Imaging Multi-Mode Reader (BioTek, Winooski, VT, USA). Data are mean ± SE of minimum four independent experiments performed in technical quadruplicates. Statistical analysis was performed with Origin Pro 9.1G (OriginLab, Northampton, USA) applying one-way ANOVA with Fisher test for pairwise comparison (threshold value *p* < 0.05).

## Results

### Influence of ATXII on intestinal cell viability

**Fig. 1:**
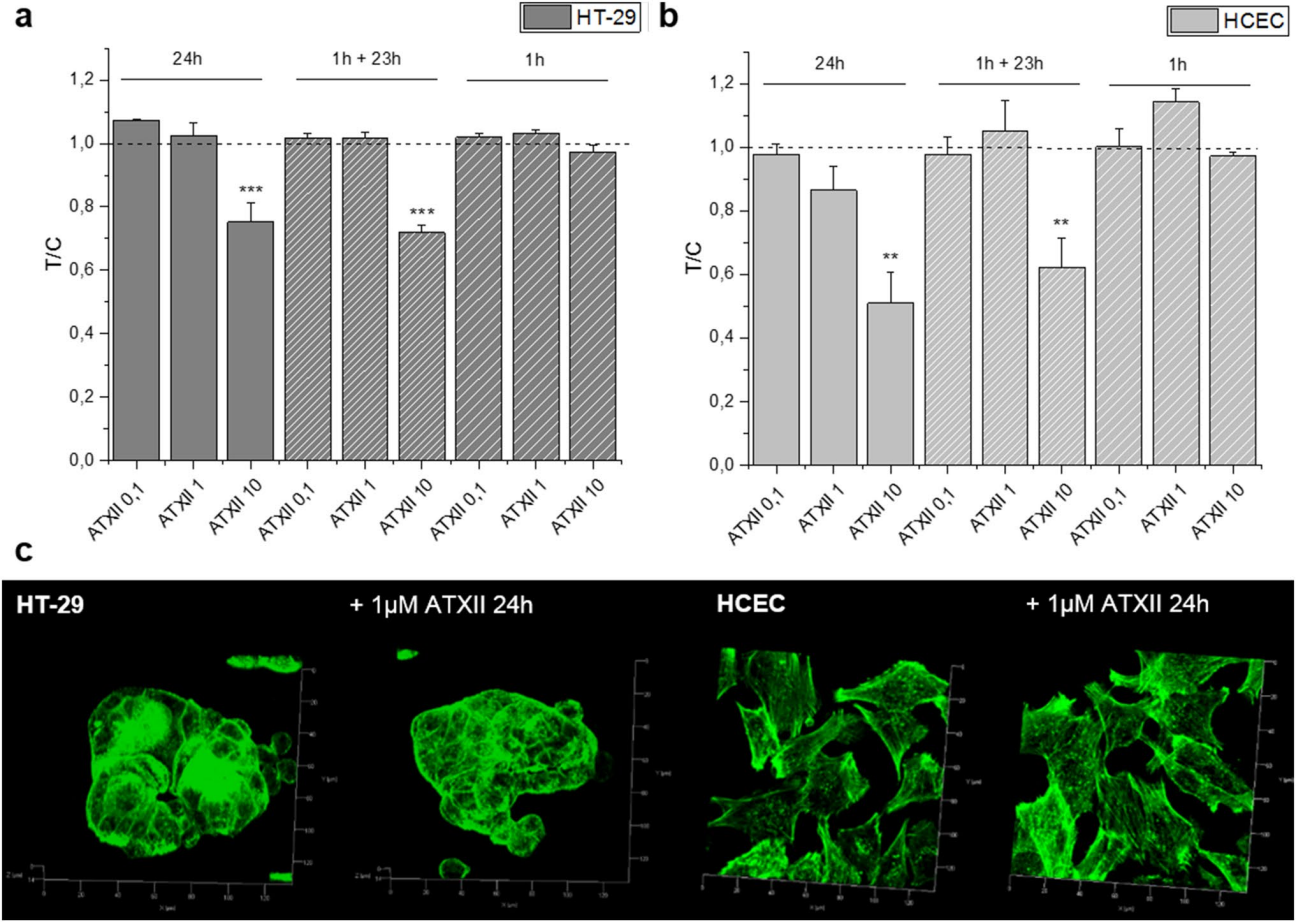
Cytotoxic potential of ATXII on intestinal cells: **a** HT-29 cells and **b** HCEC. **c** Appearance of the actin cytoskeleton in the presence or absence of ATXII (x-y axes segmentation 20 μm). Data (**a**, **b**) are presented as T/C taking as reference the solvent controls and expressed as the mean ± SE of *n* ≥ 3 independent experiments. **p < 0.01, ***p < 0.001 express significant difference in comparison to controls (one-way ANOVA and Fisher test)

To verify the potential of ATXII to induce cell damage/death, SRB experiments were performed. In HT-29 cells and HCEC (Fig. 1a, b, respectively), ATXII triggered a concentration-dependent cytotoxicity, measured as decrease of the SRB signal. Moreover, to verify the potential for reversibility of the lesions mediated by ATXII at intestinal level, recovery experiments were also included in the experimental layout. Accordingly, 1-h incubation with ATXII followed by washout and 23 h of recovery in toxin-free medium elicited the same toxicity triggered by 24 h of continuous incubation with the compound (Fig. 1a, b). The initial cytotoxicity assessment allowed also to identify 1 µM as the No Observed Effect Level both in terms of toxicity as well as at ultrastructural level, where no alteration of the organization of the actin cytoskeleton was observable (Fig. 1c).

### Influence of ATXII on the intracellular redox status of intestinal cells

**Fig. 2:**
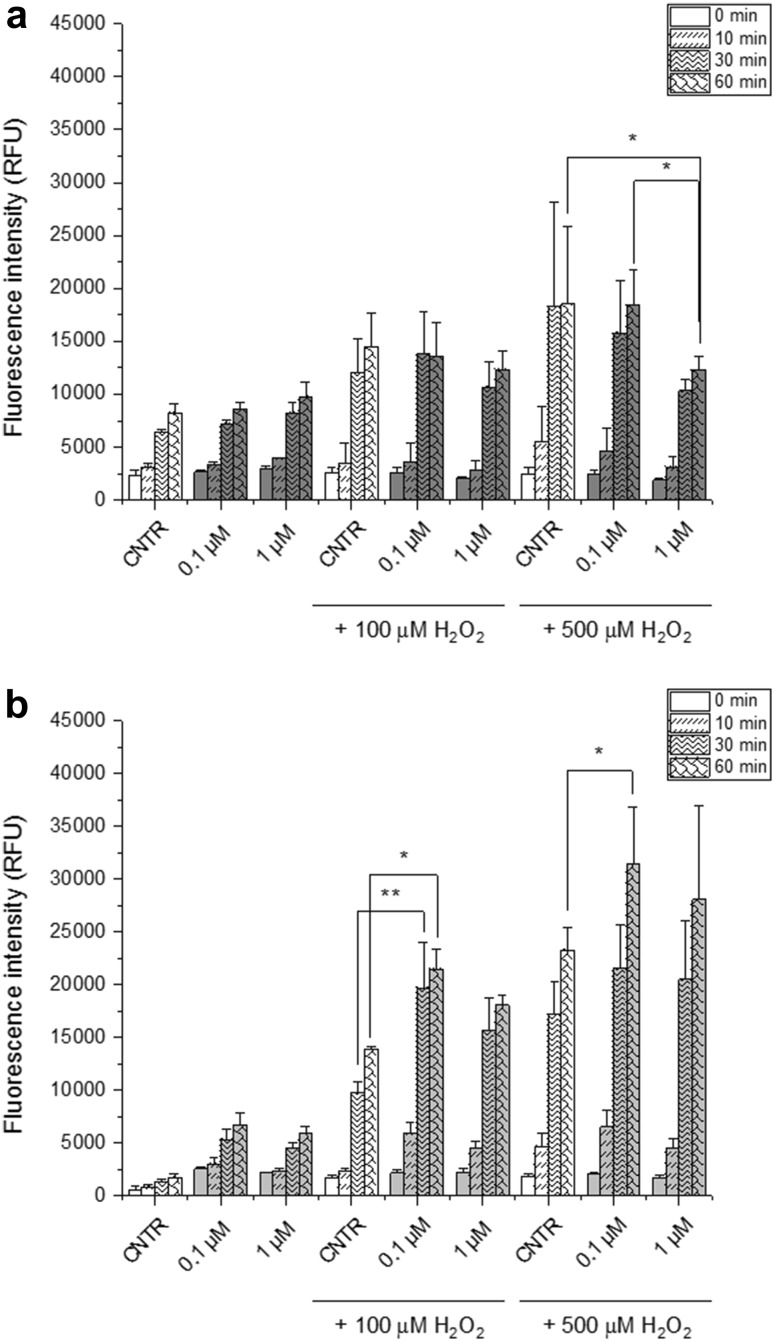
Influence of ATXII on intracellular ROS of HT-29 cells **a** and HCEC **b** measured with DCF-DA assay. Data are presented as the mean ± SE of *n* ≥ 3 independent experiments performed in quadruplicates. *p < 0.05, **p < 0.01 express significant differences in comparison to controls (white, one-way ANOVA and Fisher test)

Since 1-h exposure to ATXII was sufficient to trigger irreversible cytotoxic effect in intestinal cells, experiments were performed to verify the effect of the toxin on the ability of the epithelium to react with ROS formation. For this reason, cells were incubated with ATXII for 1 h and subsequently challenged with H_2_O_2_. Pre-incubation of HT-29 cells with ATXII (0.1 and 1 µM) moderately decreased the intracellular ROS signal triggered by H_2_O_2_ challenge (500 µM, 60 min incubation; Fig. 2a). On the other side, non-transformed cells, increased the intracellular level of ROS in response to H_2_O_2_ challenge (100 µM, 30 and 60 min incubation; 500 µM, 60 min incubation; Fig. 2b) when pre-incubated with the toxin (ATXII 0.1 µM).

### Effect of ATXII on the ability of intestinal cells to respond to mechanical stimulation

**Fig. 3:**
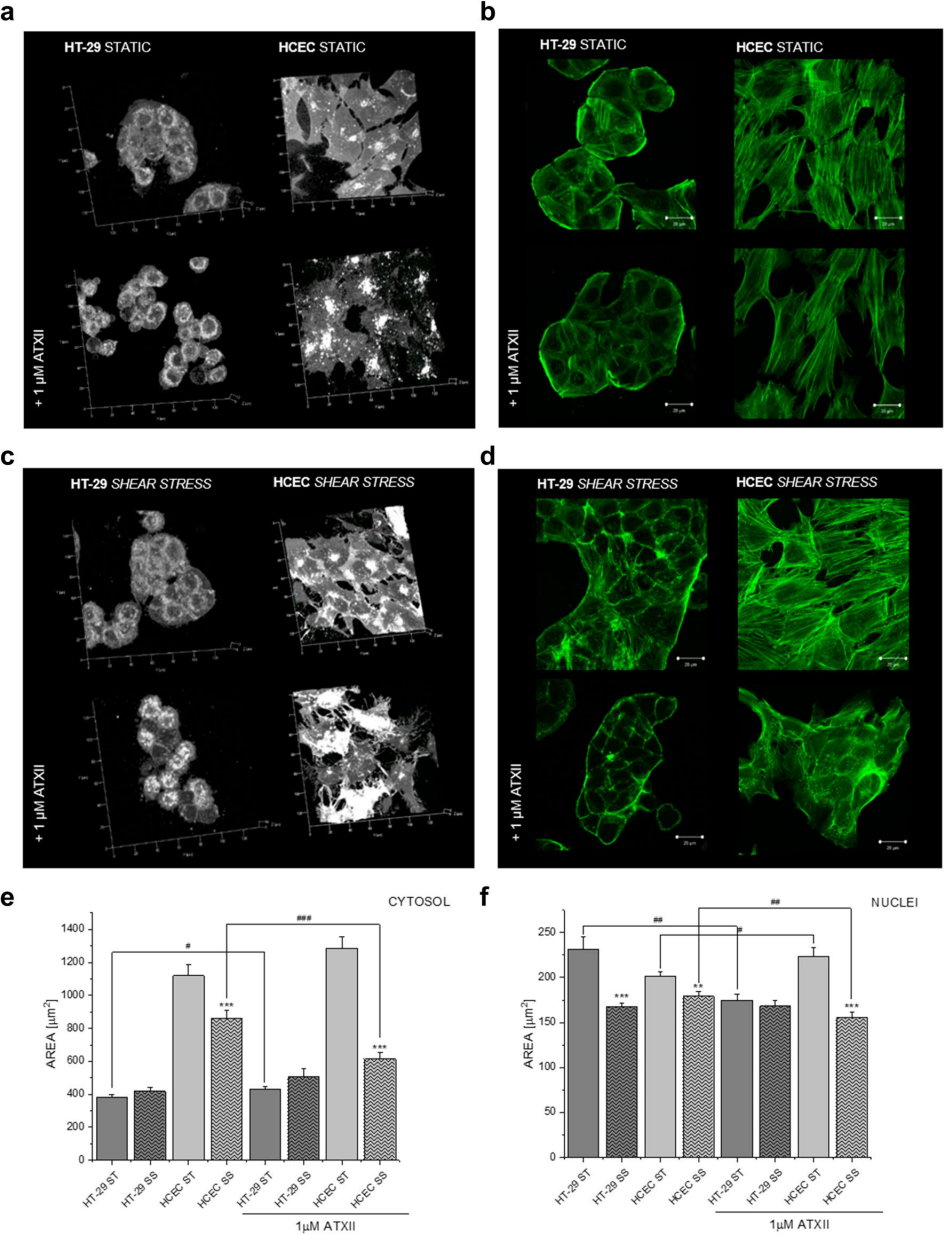
Morphological appearance of HT-29 cells and HCEC in static conditions (**a, b**) or in the presence of shear stress (**c, d**; 1 dyn/cm^2^). Appearance of the cellular membrane (gray, live cell imaging **a, c**; x-y axes segmentation 20 μm) and actin cytoskeleton (green, **b, d**; scale bars 20 μm) of the HT-29 cells and HCEC in the presence or absence of ATXII (1 µM). Quantification of cytosolic area (**e**) and nuclear area (**f**). Static conditions (ST, full bars); shear stress (SS, striped bars). Asterisk indicates significant difference in comparison to static conditions (**p < 0.01 and ***p < 0.001, Student’s *t* test; *n* > 30 cells from at least four different optical fields). ^#^Significant difference in comparison to toxin treatment (^#^*p* < 0.05; ^##^*p* < 0.01 and ^###^*p* < 0.001, Student’s *t* test; *n* > 30 cells from at least four different optical fields). (Color figure online)

Since exposure to ATXII altered the ability of intestinal cells to respond to external challenges, further experiments were performed to verify the ability of intestinal cells to respond to mechanical stimulation. In fact, in the intestinal compartment, cells are stimulated by shear stress on the luminal side. To this aim, HT-29 cells and HCEC were incubated in the presence of shear stress (1 dyn/cm^2^) for 1 h with or without toxin. Live cell imaging performed immediately after the end of the incubation revealed that in static conditions cells underwent no morphological changes (Fig. 3a); however, for HCEC, a progressive alteration of the plasma membrane was observable. Moreover, actin cytoskeleton visualization confirmed the absence of direct effects of ATXII on the morphology of both cell types in static conditions (Fig. 3b). On the contrary, combination of shear stress with the toxin altered the morphology of the cell membrane and the actin cytoskeleton (Fig. 3c, d). In addition, HCEC and HT-29 cells presented differential morphological remodeling in response to shear stress and/or to incubation with ATXII. Shear stress stimulation reduced the cytosolic area of HCEC and the concomitant presence of ATXII further enhanced this effect (Fig. 3e). In HT-29 cells the nuclear area was significantly reduced by the mechanical stimulation, as well as by the incubation with the toxin (Fig. 3f). Shear stress stimulation reduced also the nuclear area of HCEC and the concomitant presence of ATXII further enhanced this effect (Fig. 3f).

**Fig. 4:**
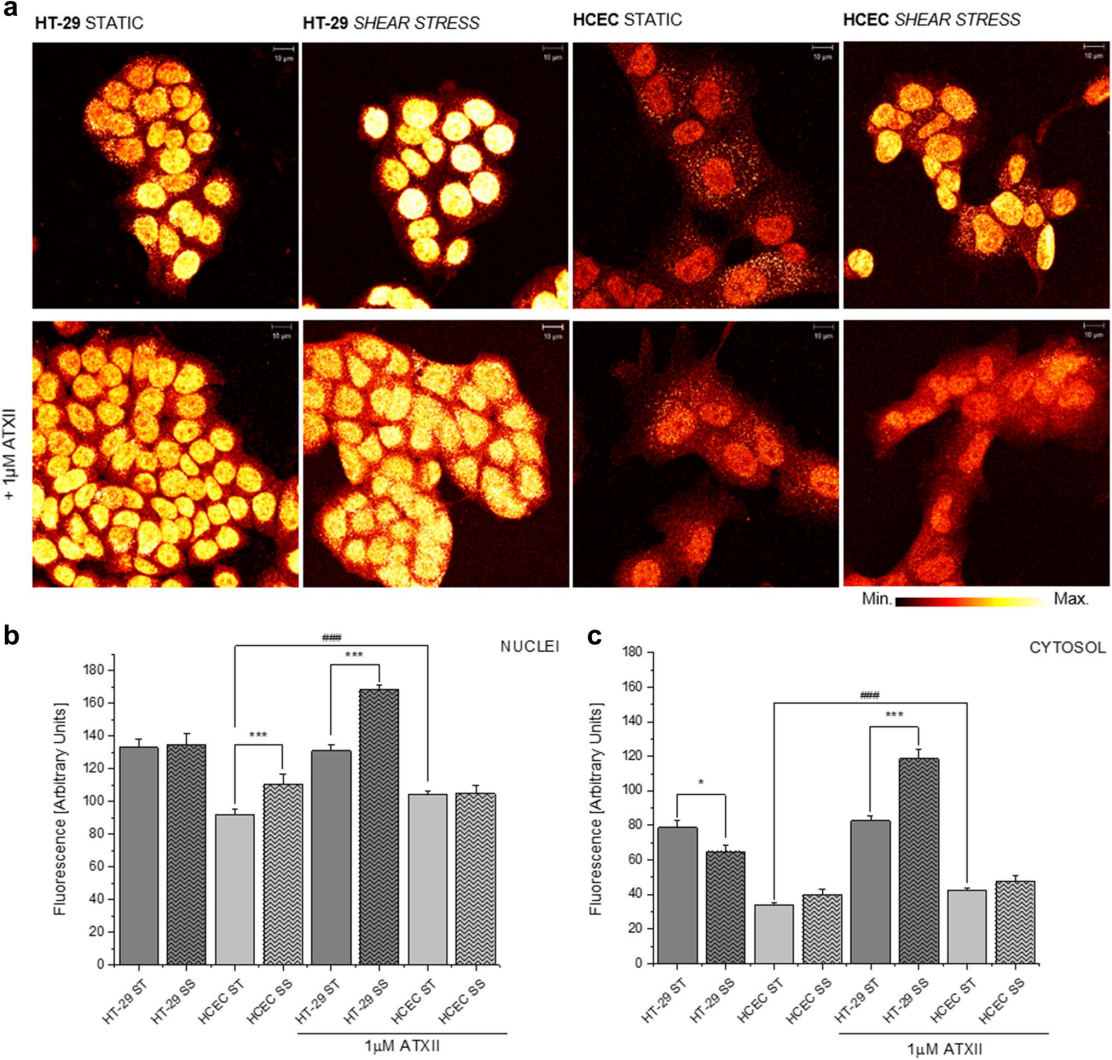
Combinatory effect of 1 µM ATXII and shear stress (1 dyn/cm^2^) on the Nrf2 (red to white depiction; scale bars 10 μm) localization in intestinal cells (**a**), quantification of the immunofluorescence localization of Nrf2 in the nuclear compartment (**b**) and cytosolic compartment (**c**). Static conditions (ST, full bars); shear stress (SS, striped bars). Asterisk indicates significant difference in comparison to static conditions (*p < 0.05; **p < 0.01 and ***p < 0.001, Student’s *t* test; *n* > 30 cells from at least four different optical fields). ^#^Significant difference in comparison to toxin treatment (^#^*p* < 0.05; ^##^*p* < 0.01 and ^###^*p* < 0.001, Student’s *t* test; *n* > 30 cells from at least four different optical fields). (Color figure online)

Moreover, since both shear stress and ATXII are able to modulate the Nrf2/ARE pathway (Chen et al. 2003; Healy et al. 2005; Jarolim et al. 2017), the localization of the transcription factor was also monitored (Fig. 4a). Quantification of the nuclear signal of Nrf2 appeared to be influenced by shear stress in HCEC, as well as by the incubation with the ATXII in static conditions (Fig. 4b). HT-29 cells appeared to be most sensitive to the combination of the shear stress and toxin with highest increase of the signal with the combination of the two stimulations (nuclei and cytosol; Fig. 4b, c). In the absence of ATXII, Nrf2 detection decreased in the cytosolic compartment of HT-29 upon shear stress stimulation (Fig. 4c).

### Impact of ATXII on the migratory ability of intestinal cells

**Fig. 5:**
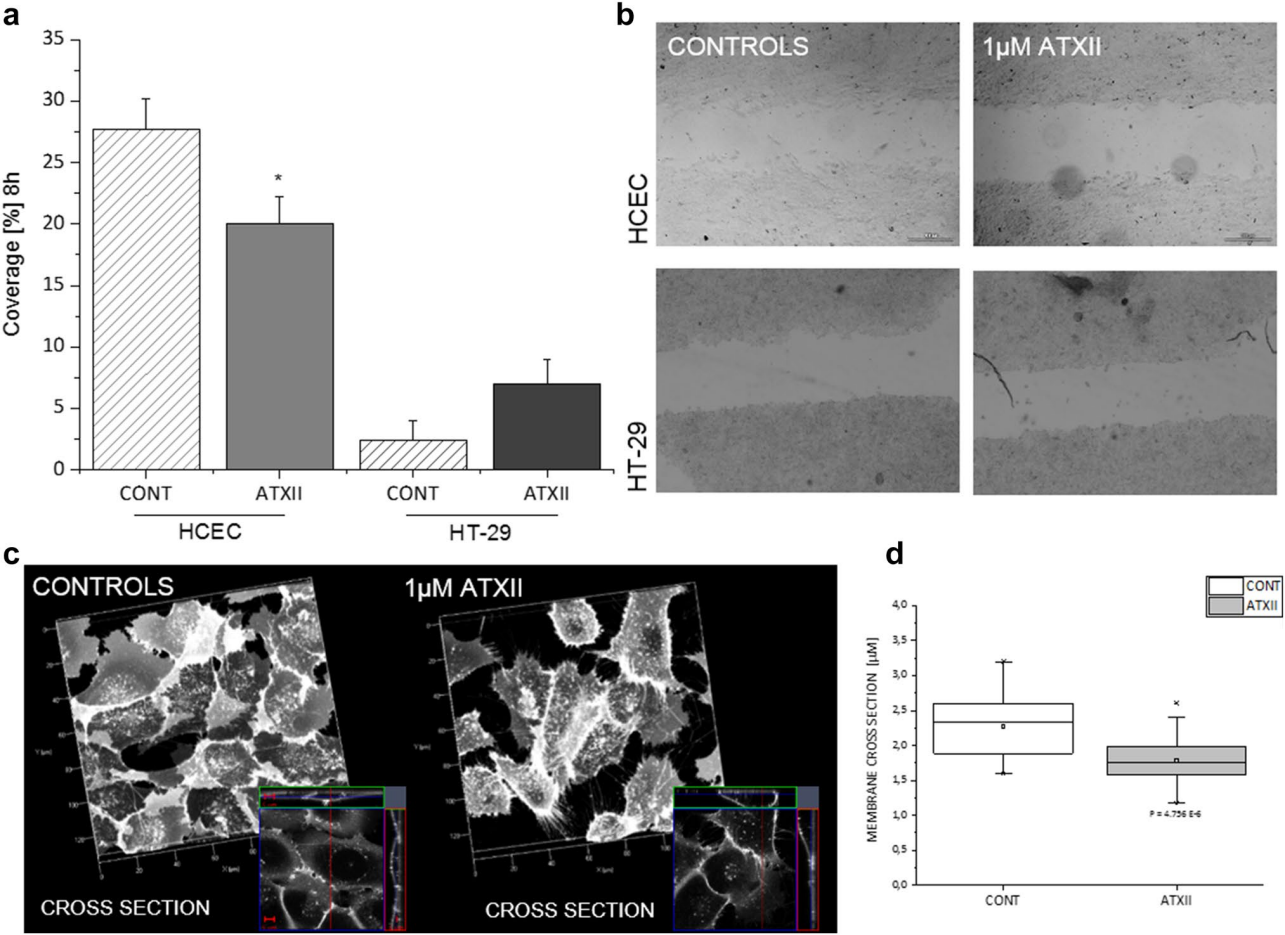
Effect of ATXII on migratory ability of HCEC and HT-29 cells. **a** Percentage (%) of gap coverage after 8 h. **b** Representative images of the appearance of the gap after 8 h (scale bars 300 μm). Data are mean ± SE of *n* > 3 independent experiments (*p < 0.05, Mann–Whitney test). **c** Representative appearance of the cell membrane of HCEC in control conditions or after 1-h incubation to 1 µM ATXII followed by 23 h of recovery in toxin-free medium (x-y axes segmentation 20 μm). **d** Quantification of the appearance of the membrane thickness in control HCEC (white box), or after 1-h incubation to 1 µM ATXII followed by 23 h of recovery in toxin-free medium (gray box); data are mean ± SE of *n* = 35 cross sections from nine different optical fields (*p* 4.756 E-6, Student’s *t* test)

To verify the impact of ATXII on the migratory ability of intestinal cells, a gap closure assay was performed. One-hour exposure to a sub-toxic concentration of ATXII (1 µM) significantly reduced the migratory ability of HCEC (Fig. 5a, b). In contrast, a tendency toward the increase of the migratory ability of HT-29 cells was observed (Fig. 5a, b). Moreover, 1-h incubation with ATXII was sufficient to alter the appearance of the thickness of the cell membrane of HCEC (Fig. 5c, d).

### Impact of ATXII on membrane fluidity of intestinal cells

**Fig. 6:**
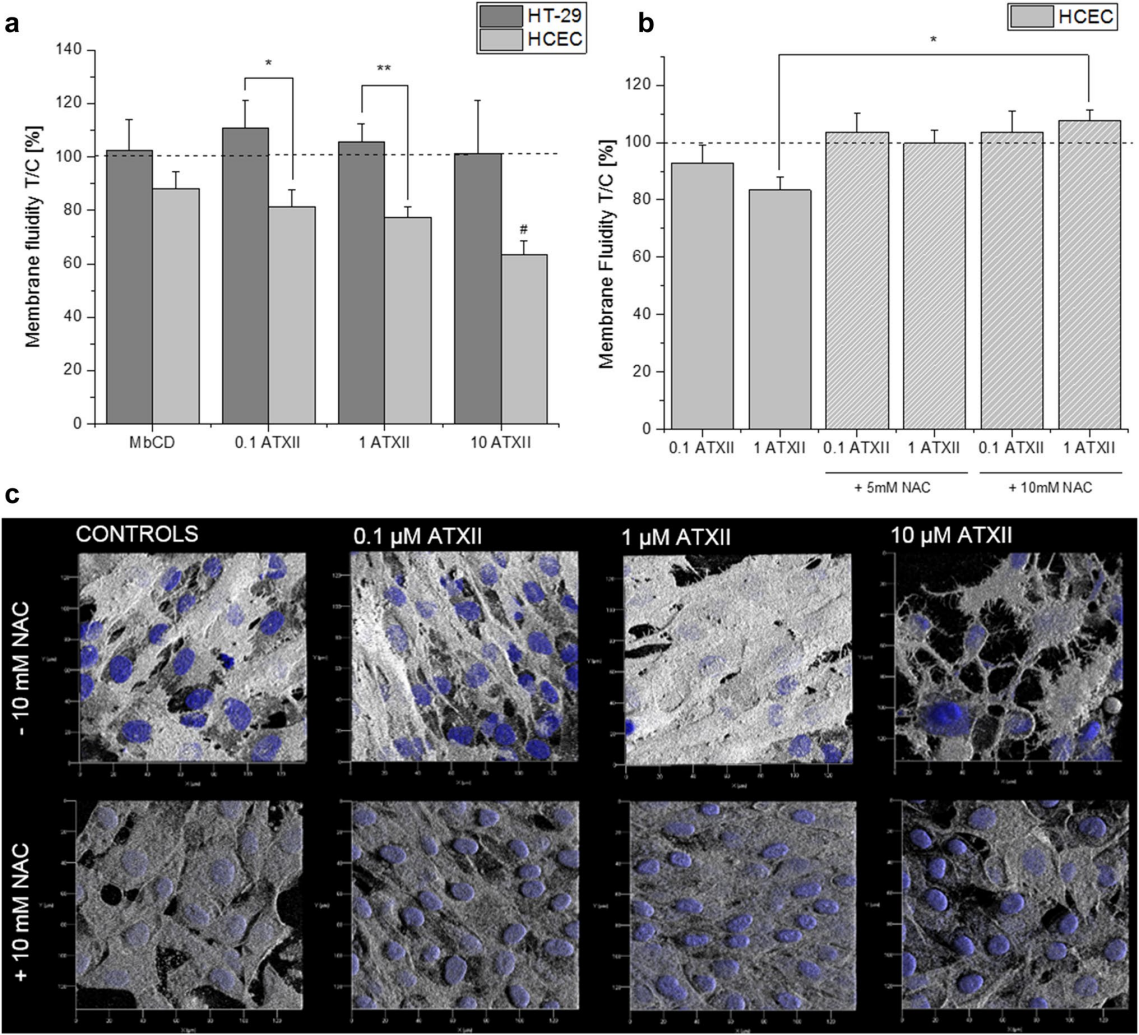
Effect of ATXII on the membrane fluidity of HT-29 cells and HCEC (**a**). Effect of *N*-acetylcysteine (NAC) on the alteration of the membrane fluidity triggered by ATXII on HCEC (**b**). Data are expressed as mean ± SE of *n* = 4–6 independent experiments performed in quadruplicate; *p < 0.05, **p < 0.01 express significant difference between the different incubation conditions, ^#^*p* < 0.05 expresses significant difference in comparison to controls. **c** Appearance of the membrane of HCEC (CellMask gray; Hoechst 33258 cell nuclei blue; x-y axes segmentation 20 μm) after incubation with ATXII in the presence or absence of NAC (10 mM). (Color figure online)

To verify whether the effect of ATXII could be sustained by an alteration of the membrane functionality, membrane fluidity of HT-29 and HCEC was measured. ATXII decreased the membrane fluidity of HCEC in a concentration-dependent manner. No significant alteration was observed for HT-29 cells (Fig. 6a). Moreover, the effect on the HCEC was reverted by co-incubation with the antioxidant N-acetylcysteine (NAC; Fig. 6b). Similar effect of NAC was confirmed also on the morphology of the membrane of HCEC (Fig. 6c).

## Discussion

Continuous movement of the intestinal content determines a very high dynamic environment in the organ lumen. As a result, the length of the exposure of single cells to toxicants can be very variable and is generally combined to movement, i.e., with physical stimulation. To mimic this scenario, the potential for reversibility of the toxic effect upon removal of the insult is a crucial step toward the characterization of the toxic insult in vitro. Interestingly, 1-h exposure to the food contaminant mycotoxin ATXII, followed by 23 h of recovery in toxin-free medium triggered in both non-transformed and cancer cells the same toxicity in comparison to 24 h of continuous incubation (Fig. 1). Moreover, 1-h incubation with the toxin significantly altered the capability of intestinal cells to respond to oxidative challenge triggered by H_2_O_2_ (Fig. 2). However, in colon carcinoma cells pre-incubation with the toxin decreased the response to H_2_O_2_, whereas in non-transformed cells (HCEC) the intracellular ROS response triggered by the pro-oxidant was increased. In agreement, many studies report that cancer cells present more elevated resistance to oxidative stress in comparison to non-tumorigenic counterparts (Van der Paal et al. 2016). The differential behavior of HT-29 and HCEC finds support in several works describing alteration of redox signaling/behavior in association with cancer progression (Trachootham et al. 2009). It is in fact well known that cancer cells often present elevated levels of ROS (Toyokuni et al. 1995; Trachootham et al. 2009). This phenomenon is generally associated with the metabolic changes occurring in cancer cells and it is often accompanied by an increase in the antioxidant capacity in tumor cells (Gorrini et al. 2013; Panieri and Santoro 2016). In this respect, a major role is played by the alteration of the Nrf2/ARE pathway, with more efficient activation of the system as “self-defense” mechanism of tumor cells and metabolic reprogramming (Iida et al. 2004; Krajka-Kuzniak et al. 2016; Sporn and Liby 2012; Taguchi and Yamamoto 2017). For a better comprehension of the molecular mechanism triggered by short-time exposure to ATXII, further experiments were performed combining the exposure to the toxin with biomechanical stimulation. This approach not only combined the exposure to the mycotoxin to a more physiological environment (Blutt et al. 2017; Kim et al. 2012), but also provided an important perspective for the comprehension of the Nrf2 regulation at intestinal level. Nrf2 translocation/activation is well known to be dependent on shear stress stimulation, playing a crucial role in endothelial cell homeostasis (Chen et al. 2003; Healy et al. 2005; Hosoya et al. 2005). However, the importance of the factor in relation to biomechanical stimulation at intestinal cell level is largely unknown. Incubation with 1 µM ATXII was combined with shear stress stimulation protocol (1 dyn/cm^2^) and compared with the incubation in static environment. Previous studies already demonstrated that this concentration of toxin does not usually trigger oxidative stress or significant alteration of the Nrf2 localization in HT-29 cells (Jarolim et al. 2017), as well as not being directly cytotoxic for both cell types (Fig. 1). Incubation of ATXII in static condition triggered a significant increase of the immunolocalization of Nrf2 in HCEC in both cytosolic and nuclear compartments, but no effect on HT-29 cells. Interestingly, the biomechanical stimulation protocol triggered in HCEC a prominent remodeling, decreasing the adhesion (surface area) of the cells to the substrate, as well as the nuclear area (Fig. 3a–d). With the combination between chemical stimulation (1 µM ATXII) and physical stimulation (shear stress, 1 dyn/cm^2^), HCEC underwent the most severe morphological alteration of the area and of the actin appearance. This observation was accompanied by a prominent alteration of the appearance of the plasma membrane (Fig. 3a, c). In comparison to HCEC, HT-29 cells presented different reactivity to physical stimulation, thus being characterized by a more compact structure with the formation, upon stimulation, of stress fibers (Fig. 3d), but no alterations to the average cytosolic area (Fig. 3e). In line, less morphological alteration was observable in the 3D rendering of the membrane structure of the cancer cells (Fig. 3a, c). In parallel, decrease of the Nrf2 localization was observed in the cytosolic compartment, possibly sustained by the tight connection between the transcription factor and actin cytoskeleton (Kang et al. 2004) (Fig. 4). Notably, the nuclear area was strongly reduced upon biomechanical stimulation of colon carcinoma cells possibly suggesting the high plasticity of this compartment (Fig. 3f). Interestingly, in tumor cell combination of ATXII with shear stress elicited the highest response in terms of Nrf2 activation, sustaining the idea of this signaling cascade being particularly enhanced and sensitive in tumor cells (Shibata et al. 2008). Moreover, the idea that cancer cells might possess an altered sensitivity/dependence from biomechanical stimulation is now commonly accepted (Broders-Bondon et al. 2018). The combination of physical and chemical stimulations increased the nuclear localization of the transcription factor Nrf2 (Fig. 4) suggesting, as for the DCF-DA experiments (i.e., H_2_O_2_ challenge; Fig. 2a), that multiple stressors could more efficiently trigger the antioxidant machinery of cancer cells. In line, several authors described an increased Nrf2 activation in cancer cells in response to stress as well as to anti-cancer therapy (Mitsuishi et al. 2012; Taguchi and Yamamoto 2017; Venkatraman et al. 2015). Interestingly, the potentiation of the Nrf2 signaling was not associated with any morphological difference (i.e., nuclear/cytosolic area), decreasing the chances that the signal regulation could be uniquely dependent on artefacts related to structural remodeling. As for the HCEC, the co-incubation of the toxin with shear stress hindered the intra-nuclear increase of the transcription factor normally triggered by the biomechanical stimulation per se. This observation, associated with the pronounced alteration of actin structure seems to suggest a possible loss of function related to the combinatory exposure. It was previously demonstrated that membrane fluidity plays a major role in the organization of the underlying cytoskeleton (Ayee et al. 2017), thus having crucial importance also in the response to shear stress (Liu et al. 2014). In line, it is possible to hypothesize that the loss of membrane fluidity (increased rigidity) triggered by the toxin could ultimately lead to a decreased sensitivity toward the signaling cascade connecting shear stress, membrane, actin and Nrf2 localization–translocation.

Since ATXII proved to be very efficient in altering the ability of intestinal cells to respond to biomechanical challenge, further experiments were performed to verify if the effect of 1-h exposure to the toxin could influence the migratory ability of intestinal cells, being migration essential for correct cellular turnover. Accordingly, 1-h incubation with 1 µM ATXII was sufficient to significantly hinder migration of HCEC (Fig. 5a, b), whereas no effect was seen in HT-29 cells, if a tendency toward the increase of the migratory ability. In these experimental conditions, proliferation of HT-29 and HCEC in the presence of 1 µM ATXII was comparable to that of controls (Fig. 1), thus reducing the chances that the effects on migration could be an artefact related to proliferation/cytotoxicity unbalance. Migratory abilities are directly related to Nrf2 translocation (Ciamporcero et al. 2018; Liao et al. 2015; Shen et al. 2014), and enhanced upon the activation of the signaling cascade. Interestingly, upon incubation with ATXII, an increased Nrf2 signal in both cytosolic and nuclear compartments was observed in HCEC, excluding that this pathway could play a role in the abovementioned effect of the toxin on intestinal cells (i.e., inhibition of migration). In our experimental conditions (1-h incubation, 1 µM ATXII, static), ATXII did not alter the actin cytoskeleton of HCEC (Fig. 3b), hence direct involvement of the cytoskeletal dynamics was excluded. Since the effect of the toxin on the biomechanical response of intestinal cells seemed to be more evident upon challenge (i.e., migration or shear stress stimulation), the attention was directed to the cellular interface with the extracellular environment, namely the plasma membrane. In this respect, it was previously demonstrated that variation of the membrane biophysical properties can downstream at intracellular level ultimately resulting in alteration of cellular cytoskeleton and motility (Ayee et al. 2017; Blanchard and Busik 2017; Kowalewski et al. 2015). In agreement with this hypothesis, the appearance of the membrane 3D structure of cells incubated with ATXII appeared to be less homogenous in comparison to controls and characterized by areas of uneven accumulation of the fluorescent CellMask dye (Fig. 3a). Moreover, 1-h exposure to the toxin followed by 23 h of recovery in toxin-free medium significantly modified the appearance of the cell membrane of HCEC (Fig. 5c, d). In accordance, the impact of ATXII on membrane fluidity of the two cell types was measured. As previously reported for the migration assay, cancer-derived and non-transformed cells responded differentially to the toxin, showing opposite behaviors on the membrane fluidity assay. In HCEC, membrane fluidity was decreased in a concentration-dependent fashion upon incubation with the mycotoxin. On the contrary, membrane biophysical properties of HT-29 cells were not altered by the incubation with the toxin, with the exception to a limited tendency toward the increase for the incubation with 0.1 and 1 µM ATXII. Interestingly, the effects of ATXII on the cell membrane appeared in a low concentration range, being the toxin tested routinely in vitro up to 20 µM (Vejdovszky et al. 2017b). It was recently reported that alteration of membrane fluidity could be directly correlated to cell migratory/metastatic capabilities both in vitro and in vivo (Edmond et al. 2015; Lin et al. 2017). In line, the evaluation of the oxidative status of the membrane is gaining more and more importance in the comprehension of the complex loss/gain of functional landscapes associated with the progression of cancer malignancy (Van der Paal et al. 2016), as well as representing a promising target for future therapeutic strategies (Peetla et al. 2013; Suganuma et al. 2016). According to this interpretation, also for the membrane fluidity HCEC appeared to be more sensitive to the effect of the mycotoxin in comparison to the tumorigenic counterpart. Moreover, the stronger response elicited by H_2_O_2_ challenge after incubation of HCEC with ATXII in comparison to HT-29 sustains the interpretation that the membrane of the non-transformed cells could be more sensitive, and consequently, the barrier function of the epithelial cells more fragile. Due to the presence of an epoxide group at the perylene quinone scaffold, ATXII is known to be a relatively reactive compound (Aichinger et al. 2018; Fleck et al. 2014a). In this light, it is prone not only to the activation of the Nrf2/ARE pathway (Jarolim et al. 2017), but also to direct reaction with diverse cell structures (Fleck et al. 2012; Jarolim et al. 2016). To verify if the effect of the toxin on membrane fluidity was related to oxidation/reaction processes involving the epoxide moiety of the molecule, experiments were performed in the presence of the antioxidant NAC (Oommen et al. 2016; Raza et al. 2016). NAC significantly reverted the effect of the toxin restoring the membrane fluidity to control levels, as well as restoring cell morphological alteration induced by ATXII (Fig. 6b, c). This suggests either the antioxidant capacity of the compound or a direct reaction/inactivation of ATXII mediated by NAC, due to the known SH reactivity of the mycotoxin (Fleck et al. 2014b; Jarolim et al. 2017), thus abolishing the effect of mycotoxin on the plasma membrane of HCEC.

In conclusion, HT-29 cells and HCEC responded differentially to the chemical/physical challenge. Shear stress proved to be effective in triggering the nuclear translocation of Nrf2 in intestinal cells opening new scenarios in the comprehension of the antioxidant machinery at the intestinal level, as well as contributing to delineate the differences of reactivity between cancer cells and healthy ones. Biophysical properties of the plasma membrane appeared to be crucial in mediating the physiological response of intestinal cells to the extracellular environment, as well as in mediating the response to toxicants. Cancer cells might respond differentially to food constituents and contaminants in comparison to the healthy counterparts, especially when incubated in complex scenarios with great repercussions on our comprehension of the effect of nutrition and therapy in intestinal cells.
